# The novel estrogen receptor beta agonist EGX358 and *APOE* genotype influence memory, vasomotor, and anxiety outcomes in an Alzheimer’s mouse model

**DOI:** 10.3389/fnagi.2024.1477045

**Published:** 2024-11-13

**Authors:** M. R. Schwabe, A. W. Fleischer, R. K. Kuehn, S. Chaudhury, J. M. York, D. S. Sem, W. A. Donaldson, M. J. LaDu, K. M. Frick

**Affiliations:** ^1^Department of Psychology, University of Wisconsin-Milwaukee, Milwaukee, WI, United States; ^2^Department of Chemistry, Marquette University, Milwaukee, WI, United States; ^3^Department of Anatomy and Cell Biology, University of Illinois at Chicago, Chicago, IL, United States; ^4^Department of Pharmaceutical Sciences Wisconsin and Concordia University Center for Structure-Based Drug Design and Development, Concordia University Wisconsin, Mequon, WI, United States

**Keywords:** estrogen receptor beta (ERβ), apolipoprotein E, object recognition, object placement, hot flash, Alzheimer’s disease, open field, hippocampus

## Abstract

**Introduction:**

Alzheimer’s disease (AD) prevalence and severity are associated with increased age, female sex, and apolipoprotein E4 (APOE4) genotype. Although estrogen therapy (ET) effectively reduces symptoms of menopause including hot flashes and anxiety, and can reduce dementia risk, it is associated with increased risks of breast and uterine cancer due to estrogen receptor alpha (ERα)-mediated increases in cancer cell proliferation. Because ERβ activation reduces this cell proliferation, selective targeting of ERβ may provide a safer method of improving memory and reducing hot flashes in menopausal women, including those with AD. APOE genotype influences the response to ET, although it is unknown whether effects of ERβ activation vary by genotype.

**Methods:**

Here, we tested the ability of long-term oral treatment with a novel highly selective ERβ agonist, EGX358, to enhance object recognition and spatial recognition memory, reduce drug-induced hot flashes, and influence anxiety-like behaviors in female mice expressing 5 familial AD mutations (5xFAD-Tg) and human APOE3 (E3FAD) or APOE3 and APOE4 (E3/4FAD). Mice were ovariectomized at 5 months of age and were then treated orally with vehicle (DMSO) or EGX358 (10 mg/kg/day) via hydrogel for 8 weeks. Spatial and object recognition memory were tested in object placement (OP) and object recognition (OR) tasks, respectively, and anxiety-like behaviors were tested in the open field (OF) and elevated plus maze (EPM). Hot flash-like symptoms (change in tail skin temperature) were measured following injection of the neurokinin receptor agonist senktide (0.5 mg/kg).

**Results:**

EGX358 enhanced object recognition memory in E3FAD and E3/4FAD mice but did not affect spatial recognition memory. EGX358 also reduced senktide-induced tail temperature elevations in E3FAD, but not E3/4FAD, females. EGX358 did not influence anxiety-like behaviors or body weight.

**Discussion:**

These data indicate that highly selective ERβ agonism can facilitate object recognition memory in both APOE3 homozygotes and APOE3/4 heterozygotes, but only reduce the magnitude of a drug-induced hot flash in APOE3 homozygotes, suggesting that APOE4 genotype may blunt the beneficial effects of ET on hot flashes. Collectively, these data suggest a potentially beneficial effect of selective ERβ agonism for memory and hot flashes in females with AD-like pathology, but that APOE genotype plays an important role in responsiveness.

## 1 Introduction

Women comprise nearly two-thirds of individuals in the U.S. living with Alzheimer’s Disease (AD), a majority of whom develop cognitive symptoms of the disease after age 65 (Alzheimer’s Disease and Dementia, 2024). The primary risk factors for this “late-onset” AD are advanced age, female sex, and apolipoprotein E (*APOE*) genotype (Corder et al., 1993; Riedel et al., 2016; Valencia-Olvera et al., 2023). Humans express two copies of three *APOE* alleles, ε2, ε3, and ε4, in different combinations, where risk of AD is reduced in ε2 carriers, neutral in ε3 carriers, and increased in ε4 carriers (Corder et al., 1993; Roses, 1996; Bertram, 2009). In particular, women with one or two copies of *APOE4* are at highest risk of developing AD (Valencia-Olvera et al., 2023; Bretsky et al., 1999; Altmann et al., 2014; Rajan et al., 2021).

A potential contributing factor to the increased incidence of AD in women is the loss of estrogens associated with menopause, as estrogens are important neuroprotective factors for neurons in brain regions like the hippocampus and medial prefrontal cortex (mPFC) that mediate memory and are particularly vulnerable to characteristic AD pathologies including development of amyloid plaques and neurofibrillary tangles, and loss of synapses and neurons (Beffert et al., 1999; Brann et al., 2007; Small et al., 2011; Fjell et al., 2014; Taxier et al., 2020). Indeed, older women with low serum bioavailable estrogens are more likely to develop global cognitive decline and verbal memory loss than those with higher estrogen levels (Yaffe et al., 2000; Yaffe et al., 2007; Zsido et al., 2019). Moreover, peri- and post-menopausal women exhibit greater rates of cognitive decline than men, and post-menopausal women have the highest rate of hippocampal volume loss relative to younger women and men (Mosconi et al., 2018). People experiencing hot flashes may be at particularly increased risk of cognitive decline and AD, as night-time hot flashes in women are associated with impaired verbal memory (Maki et al., 2008) and higher plasma biomarkers of amyloid plaques (Thurston et al., 2024). Although older randomized clinical trials of conjugated equine estrogen (CEE)-based therapies (e.g., Premarin with or without medroxyprogesterone acetate) suggest that treatment does not reduce AD risk in post-menopausal women over age 65, observational studies of 17β-estradiol (E_2_)-based treatments consistently report that E_2_ use reduces AD risk and brain amyloid deposition assessed via post-mortem autopsy or PiB PET scanning in middle-aged women when initiated during perimenopause or early menopause (Yaffe et al., 2000; Kantarci et al., 2016; Chen et al., 2022; Nerattini et al., 2023; Wong et al., 2023), suggesting beneficial effects of early E_2_ use.

However, effects of estrogen therapy are more complex when participants are stratified by *APOE* genotype, with some studies of older post-menopausal individuals (mean age > 65 years) reporting that treatments consisting primarily of CEE did not benefit memory or reduce dementia risk in *APOE4+* women (Yaffe et al., 2000; Burkhardt et al., 2004) or detrimentally affected cognition in *APOE4+* women (Kang et al., 2004; Kang and Grodstein, 2012). In contrast, more recent studies of younger menopausal women (mean age < 65 years) treated with E_2_ report beneficial effects on brain amyloid deposition and volumes of the hippocampus and entorhinal cortex in *APOE4* carriers only (Kantarci et al., 2016; Saleh et al., 2023), and greater changes in biomarkers of plaque accumulation in *APOE4*+ vs. *APOE4*- women (Depypere et al., 2023). Thus, mid-life initiation of E_2_-based therapies may be beneficial for cognition and reduce AD-related brain pathology even in women with one or two copies of *APOE4*, although few studies have addressed this directly, even in animal models of AD.

Another complication with the use of estrogen therapies for post-menopausal people with AD is that they can increase risks of breast and uterine cancer due to the cell proliferative effects of estrogen receptor alpha (ERα) (Ali and Coombes, 2000; Péqueux et al., 2012; Shete et al., 2023). Activation of estrogen receptor beta (ERβ) does not cause cancer cell proliferation (Jia et al., 2015), yet enhances memory formation in rodents (Boulware et al., 2013; Tian et al., 2013; Tuscher et al., 2015). ERβ is highly expressed in the hippocampus, mPFC, and hypothalamus (Shughrue et al., 1997; Milner et al., 2001; Milner et al., 2005; Almey et al., 2014), and its activation is associated with reduced Aβ levels and tau phosphorylation in the hippocampus (Tian et al., 2013; Xiong et al., 2015) Thus, highly selective ERβ agonists may improve memory and reduce AD risk in menopausal individuals both directly by boosting hippocampal and cortical function and indirectly by reducing hypothalamic-mediated hot flashes.

Currently, the selectivity of commercial ERβ agonists is generally poor (Hanson et al., 2018), prompting the need for higher-performing compounds in order to develop therapeutic treatments with minimal off-target side effects. Thus, we developed the potent and highly selective ERβ agonist EGX358, which has an EC_50_ for ERβ of 27.4 nM and is 750-fold more selective for ERβ over ERα (Hanson et al., 2018). In ovariectomized (OVX) C57BL/6 mice, acute intrahippocampal or systemic EGX358 (0.5 mg/kg) treatment facilitated consolidation of spatial and object recognition memories in hippocampus- and mPFC-dependent object placement and object recognition tasks, yet did not stimulate breast cancer cell proliferation or cause organ toxicity (Hanson et al., 2018). Similarly, 2 months of oral EGX358 treatment in OVX mice enhanced object recognition and object placement memories, as well as reduced the magnitude of a drug-induced hot flash, without adverse effects on anxiety- or depression-like behaviors or on body weight (Fleischer et al., 2021). Whether EGX358 can influence memory and hot flashes in models of AD is unknown.

Thus, the goal of the present study was to determine the extent to which long-term oral EGX358 treatment could promote memory formation and reduce the magnitude of a drug-induced hot flash in female *APOE4*- and *APOE4*+ mice using the *APOE*^+/+^/5xFAD^+/−^ (EFAD) transgenic model of AD, which models late-onset *APOE*-related AD risk by combining five amyloid precursor protein and presenilin mutations found in early-onset AD families with expression of human *APOE* gene variants (Youmans et al., 2012; Tai et al., 2017). The hippocampus and cortex of E4FAD mice exhibit elevated levels of Aβ42, oligomeric Aβ, amyloid deposition, phospho-tau, gliosis, and synaptic degeneration by 6 months of age, with exaggerated pathology in females relative to males persisting up through 18 months of age (Youmans et al., 2012; Tai et al., 2014; Liu et al., 2015; Cacciottolo et al., 2016; Balu et al., 2023). Although no animal model perfectly reproduces AD sequelae, EFAD mice are advantageous in that they model late-onset *APOE*-related AD risk in a mouse with established hippocampal and neocortical amyloid deposition, tau hyperphosphorylation, and synaptic degeneration typical of AD. This model recapitulates human clinical presentation of *APOE*-associated AD, such that female mice expressing two copies of *APOE4* (E4FAD) exhibit earlier onset of, and more advanced, disease pathology relative to E4FAD males and mice of both sexes that express two copies of *APOE3* (E3FAD) (Youmans et al., 2012; Tai et al., 2014; Liu et al., 2015; Cacciottolo et al., 2016; Balu et al., 2023). Moreover, E4FAD mice have impaired memory relative to E3FAD mice (Liu et al., 2015; Balu et al., 2023), including in object recognition and object placement tasks (Taxier et al., 2022a,b). These object memory deficits in E4FAD mice were associated with reduced dorsal hippocampus and mPFC dendritic spine density, as well as decreased dorsal hippocampus levels of the synaptic markers PSD-95 and synaptophysin and increased levels of ERα relative to E3FAD mice (Taxier et al., 2022b). Among females, acute bilateral infusion of E_2_ into the dorsal hippocampus promoted object recognition and object placement memory consolidation and increased dorsal hippocampal CA1 apical and basal dendritic spine density in E3FADs and in mice bearing one copy of *APOE3* and *APOE4* (E3/4FAD), but not in E4FADs (Taxier et al., 2022a). Although unclear why E4FAD mice are less responsive to E_2_, ERα levels, but not ERβ levels, are aberrantly high in E4FAD females (Taxier et al., 2022a,b), suggesting potential efficacy of selectively targeting ERβ in *APOE3*+ females. In support, constitutive knockout of ERβ in mice is associated with Aβ42 deposits and *APOE* accumulation in brain regions including the hippocampus and cortex (Zhang et al., 2004), suggesting that ERβ activity protects against AD-like pathologies. Because our previous work indicated that acute E_2_ infusion enhances memory in E3FAD and E3/4FAD females, the present study examined effects of long-term oral EGX358 treatment on behavioral outcomes in E3FAD and E3/4FAD females. Moreover, the translational value of these genotypes is high, as they represent the majority of *APOE* genotypes in the general population (Heffernan et al., 2016). Here, we found that EGX358 improved object recognition in both genotypes but reduced the magnitude of a drug-induced hot flash only in E3FAD females and had no detrimental effects on anxiety, body weight, or uterine weight in either genotype. Thus, highly specific ERβ agonists may be suitable and safe hormone therapies to alleviate symptoms of menopause and/or AD in females expressing one or two copies of *APOE3*.

## 2 Materials and methods

### 2.1 Subjects

*APOE*-TR^+/+^/5xFAD^+/−^ (EFAD) mice express 5 familial AD mutations (APP K670N/M671L, I716V, V717I, PS1 M146L, and L286V) under control of the neuron-specific mouse Thy-1 promoter, and express human *APOE3* or *APOE4* (Youmans et al., 2012; Tai et al., 2017). For this study, female mice were either homozygous for human *APOE3* (E3FAD) or heterozygous for *APOE3* and *APOE4* (E3/4FAD). Mice homozygous for *APOE4* (E4FAD) were not used because EFAD mice of this genotype are insensitive to the beneficial effects of E_2_ on object recognition and spatial memory or CA1 dendritic spine density (Balu et al., 2023; Taxier et al., 2022a). Given the extensive research establishing validity of the EFAD model of AD relative to wild-type conditions (Lewandowski et al., 2020) and our previous work demonstrating the efficacy of EGX358 in young wild-type female mice (Hanson et al., 2018; Fleischer et al., 2021), wild-type mice were not used in the current study. EFAD mice were bred, weaned, and genotyped at the University of Illinois at Chicago (UIC) and shipped to the University of Wisconsin-Milwaukee at 2 months of age, where they were aged to 5 months before the start of OVX surgery. Although mice of this age are still considered young adults, amyloid accumulation in EFAD mice is already striking by 4 months of age and quite significant by 6 months of age (Youmans et al., 2012; Tai et al., 2017). Our previous work with 6–7 month E3FAD and E4FAD males and females indicated that E4FAD mice of both sexes already exhibited widespread dendritic spine loss in the hippocampus and prefrontal cortex, with concomitant deficits in multiple memory tasks (Taxier et al., 2022a,b), so we sought here to intervene with EGX358 at an earlier age to model early-stage AD.

Prior to surgery, mice were housed in groups of up to 5 per cage but then were singly housed following OVX for the remainder of the study to enable treatment dosing based on each mouse’s hydrogel intake and weight. Mice were treated and behaviorally tested in 4 separate cohorts, each including approximately even numbers of E3FAD and E3/4FAD mice. Mice were maintained on a 12 h light/dark cycle (lights on a 07:00) with all procedures conducted during the light portion of the cycle. Mice received *ad libitum* access to food (Teklad Rodent Diet 8,604, Inotiv) for the duration of the study. Although typical isoflavone concentrations (daidzein + genistein aglycone equivalents) in this food range from 575 to 870 mg/kg, human diets naturally contain some soy, so this diet is more translatable than soy-free chow. Importantly, all mice received the same diet, so this food is unlikely to have contributed to observed treatment or genotype effects. Mice were provided with *ad libitum* access to water up until 2 weeks after surgery, at which point water bottles were removed and all hydration was provided via hydrogel (ClearH20) containing vehicle or EGX358 (see below for dosing information). Hydrogel consumption was measured daily and replenished as needed. Protocols and procedures followed the National Institutes of Health Guide for the Care and Use of Laboratory Animals and were approved by the University of Wisconsin-Milwaukee Institutional Animal Care and Use Committee.

### 2.2 General experimental design

**Figure 1:**
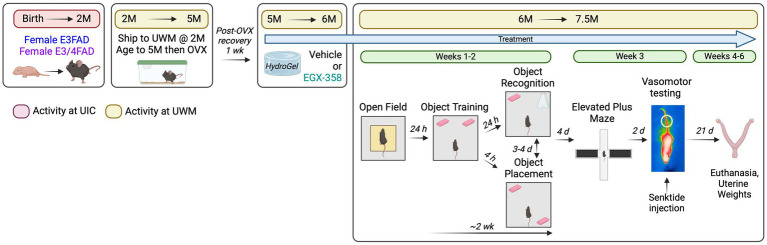
General experimental design. Female E3FAD and E3/4FAD mice were bred at the University of Illinois at Chicago (UIC) and then shipped to the University of Wisconsin-Milwaukee (UWM) at 2 months of age, where they were housed without any manipulations until 5 months of age. At 5 months, mice were ovariectomized and 1 week later began daily oral treatment with vehicle (10% DMSO) or EGX358 (10 mg/kg/day) dissolved in hydrogel. Following 4 weeks of treatment, mice were tested in open field, object placement, and object recognition tasks. Four days after the final memory test, mice were tested in an elevated plus maze, followed 2 days later by vasomotor testing. During vasomotor testing, baseline tail skin temperature was recorded via infrared imaging for 10 min followed by a single subcutaneous injection of senktide (0.5 mg/kg) and then 20 min of post-injection tail skin temperature monitoring. Mice were euthanized 3 weeks after vasomotor testing for dissection and recording of uterine weights. Created in BioRender. Frick, K. (2024) https://BioRender.com/g76j299

A schematic of the study’s overall experimental design is shown in Figure 1. As described in section 2.1, mice were bred at UIC and sent to UWM at 2 months of age where they were matured to 5 months of age to allow for the development of amyloid deposition, elevated phospho-tau, gliosis, and synaptic degeneration (Youmans et al., 2012; Tai et al., 2014; Liu et al., 2015; Cacciottolo et al., 2016; Balu et al., 2023). At 5 months of age, mice were OVX and 1 week later began oral treatment with vehicle (dimethylsulfoxide, DMSO) or EGX358 (10 mg/kg/day) mixed into hydrogel water gel for animal hydration. Following 4 weeks of initial treatment, mice began behavioral testing, through which daily treatments continued. The month-long pre-training treatment provided ample time for EGX358 to exert long-term effects on brain function prior to behavioral assessment; treatment continued through the study until tissue collection to assess effects of chronic treatment on behavioral and physiological outcomes. After initial pre-treatment, mice were tested in the open field to assess locomotor activity and anxiety-like behaviors, followed 24 h later by the start of object placement and object recognition testing. Object placement and object recognition tests were conducted on separate days 3–4 days apart for an individual mouse, the order of which was counterbalanced within and between groups. Together, open field and testing in both object tasks lasted about 2 weeks. Four days after the conclusion of memory testing, anxiety-like behaviors were evaluated in the elevated plus maze, followed 2 days later by assessment of vasomotor reaction to a single drug-induced hot flash. Together, plus maze and hot flash testing lasted about 1 week. Mice were euthanized 3 weeks later for measurement of uterine weights.

### 2.3 Ovariectomy surgery

Mice were single-housed and provided with ¼ of a carprofen (Rimadyl, Bioserv) tablet (2 mg/full tablet) in their cage the day prior to surgery. At the start of surgery, mice received a 5 mg/kg subcutaneous dose of Rimadyl for pain management. They were then anesthetized with isoflurane in 100% oxygen (5% for induction, 2–3% for maintenance). Briefly, two dorsal incisions were first made in the skin over the pelvic bones and underlying muscle walls. The ovaries and oviducts were isolated, ligated at the tip of the uterine horn, tied off with nylon monofilament (Monomid), and removed. The muscle walls were then sutured with chromic gut, and incision sites closed with wound clips, which were removed 6–8 days after surgery (Lewis et al., 2008). Mice received additional ¼ carprofen tablets 12 and 36 h after surgery for postsurgical analgesia and were given 1 week to recover prior to the start of drug administration.

### 2.4 Drug preparation and administration

Long-term oral vehicle and EGX358 treatments were dissolved in 10% DMSO and delivered via HydroGel (ClearH_2_O), which served as the sole source of hydration. Hydrogels were prepared separately for each mouse as per manufacturer instructions. Briefly, to make an aliquot for one mouse, 50 g of gel was melted in a 50 mL Falcon tube in a 60°C water bath for 10 min and then vehicle or EGX358 solution was injected into the gel using aseptic technique, and the tube shaken by hand and then vortexed vigorously for up to a minute. Our pilot testing with a blue dye injected into the melted gel at the same volume as dissolved compound filled the gel in a completely homogenous manner. Prepared gel was placed in a plastic cup affixed to the wall of the cage and mice were allowed to freely consume the gel which was replenished daily between 8 am and 12 pm. Gel was made fresh for each mouse every 2–3 days, based on the mouse’s weight and average gel consumption across the previous week. Mice were first acclimated to plain hydrogel consumption without treatment for 3 days. Mice quickly learned to consume the gel and readily ate the entire gel presented to them, resulting in normal levels of hydration. Mean consumption (g/day) was calculated and used to determine the concentration of drug required to achieve 10 mg/kg/day EGX358 dissolved in 10% DMSO. This dose was based on our previously published work in which EGX358 was administered via oral gavage at a dose of 0.5 mg/kg/day (Hanson et al., 2018; Fleischer et al., 2021) and unpublished observations from our lab that estrogen compound dosing should be increased by approximately 20x for HydroGel administration. As a control, vehicle gels were mixed with the same amount of 10% DMSO relative to volume of gel consumption. Mice were weighed weekly (g), and gel consumption was recorded daily, both of which were used to adjust the amount of EGX358 in DMSO to achieve the desired 10 mg/kg/day dose.

To chemically induce a rapid and transient rise in tail skin temperature meant to mimic a hot flash, the neurokinin-3 tachykinin receptor (NK3R) agonist senktide (Tocris Biosciences) was injected just once after a 10 min baseline recording period. Senktide was dissolved in 0.9% saline and injected subcutaneously at a dose of 0.5 mg/kg (5 mL/kg) (Fleischer et al., 2021; Krull et al., 2017).

### 2.5 Open field, object recognition, and object placement tasks

Following 4 weeks of daily treatment with hydrogel, mice were habituated to an open field for 5 min/day for 2 days to prepare them for subsequent object recognition and object placement training and testing (Fleischer et al., 2021). Each day, mice were placed into the lower center of an empty white box (60 cm × 60 cm × 47 cm) divided into a 5 × 5 square grid. During the first day of habituation, exploratory behaviors were recorded and quantified automatically by Any-maze software (Stoelting), which tracked the center of the mouse’s body (Hanson et al., 2018; Fleischer et al., 2021). Time (s) spent in the center zone (innermost 9 squares) and outer zone (outermost 16 squares) were measured as indications of exploration and anxiety-like behavior (more center time indicates less anxiety-like behavior) (La-Vu et al., 2020). Path length (m) and average speed (m/s) were recorded to assess locomotor function and measure potential baseline effects of treatment or genotype on performance factors that might influence object memory results. Our previous work showed no differences between OVX E3FAD and E4FAD mice on these measures (Taxier et al., 2022c), suggesting no effect of *APOE4* homozygosity on anxiety-like or explorative behaviors in the open field.

Twenty-four hours after the second habituation session, mice were trained in object recognition (OR) or object placement (OP) tasks, the order of which were counterbalanced within each group. OR and OP were chosen to measure object recognition and spatial memory, respectively, as both depend on the integrity of the dorsal hippocampus and mPFC (Eichenbaum, 2017; Tuscher et al., 2018; Sauer et al., 2022). Further, our laboratory has shown repeatedly that OR and OP are sensitive to the memory-enhancing effects of E_2_ (Koss et al., 2018; Kim et al., 2019; Gross et al., 2021) and ERβ agonism (Boulware et al., 2013; Hanson et al., 2018; Fleischer et al., 2021) in OVX wild-type and the memory-enhancing effects of E2 in OVX EFAD mice (Taxier et al., 2022a). In the training phase, mice were first accustomed to the empty open field box for 2 min and then returned to their home cage, during which time two identical objects were placed near the northeast and northwest corners of the box. Mice were then returned to the box and allowed to freely explore the objects until they had accumulated 30 s of object exploration (defined by nose contact with the objects) or until 20 min had elapsed. Those that did not reach this criterion were not advanced to testing right away, but rather were given a second training session with different object a few days later, as our experience indicates that some mice overcome a hesitancy to explore the objects when given a second chance to do so. As described below in section 2.9, all mice that underwent this second training session met the 30 s criterion so were advanced to testing.

Testing occurred 24 h later for OR and 4 h later for OP, which are timepoints at which acute E_2_ treatment previously enhanced memory relative to vehicle treatment in OVX E3FAD and E3/4FAD mice (Taxier et al., 2022a). During testing, one training object was replaced with a novel object (OR) or moved to the southwest or southeast corner (OP), and mice were again allowed to accumulate 30 s of total exploration. The time spent with novel or moved object, respectively, as well as time to accumulate 30 s of exploration, were quantified. Because mice have an inherent preference for novelty, those who remember the identity and location of the training objects should spend significantly more time than chance (15 s) with the novel and moved objects. Interactions with the objects were manually scored in ANY-maze (Stoelting Co.) by researchers blinded to genotype and treatment groups. To assess non-mnemonic performance factors during training, distance traveled (m) and velocity (m/s) were also analyzed. Between each mouse, the open field was cleaned with 70% ethanol.

### 2.6 Elevated plus maze

As an additional measure of anxiety-like behaviors, elevated plus maze (EPM) testing was performed as described previously (Fleischer et al., 2021). Briefly, the EPM apparatus consisted of two open arms (30 cm x 5 cm) constructed from white Plexiglass with a clear lip (0.5 cm) attached to the sides to prevent mice from falling, two closed arms (30 cm x 5 cm x 15 cm) made of black Plexiglass walls, and a gray Plexiglass floor. The four arms converged in the center zone. Mice were placed in the center of the maze facing the open arm furthest from the experimenter and allowed to freely explore for 10 min. Entry into the open or closed arms was defined as the presence of all four paws within the arm; mice were considered to be in the center zone when at least one paw was outside of an arm. Number of entries into and time spent in the center zone and in each arm were quantified manually in Any-maze by a researcher blind to treatment and genotype. The EPM apparatus was cleaned with mild soap and water between each test.

### 2.7 Drug-induced vasomotor response

A primary indicator of hot flashes is vasodilation, which can be measured in rodents by infrared imaging of tail skin temperature (T_Skin_), which increases to release excess body heat. Injection of the NK3R agonist senktide induces rapid and transient neurokinin B-mediated vasodilation and is used to study the neural mechanisms underlying hot flashes (Krull et al., 2017; Krajewski-Hall et al., 2017; Padilla et al., 2018). We previously reported that long-term daily oral gavage with EGX358 reduced T_Skin_ in response to acute subcutaneous senktide injection in OVX wild type mice (Fleischer et al., 2021). Here, on the day of acute senktide injection (0.5 mg/kg), mice were placed in their home cage in a secondary container (10 in x 18 in x 10 in) beneath a thermal camera (E8, FLIR, Wilsonville, OR) for 1 h to acclimate to the novel environment and testing room. After acclimation, the home cage lid was removed and continuous thermal imaging was initiated. T_Skin_ was recorded for the next 10 min to establish a baseline and to minimize effects of stress or altered behavior due to cage top removal. Next, a researcher removed the mouse from its home cage and administered a subcutaneous injection of senktide. Upon returning the mouse to its cage, T_Skin_ was recorded for another 20 min. The cage top was then replaced and the mouse returned to the colony room.

T_Skin_ was quantified using FLIR Tools+ software according to previous work (Fleischer et al., 2021; Krull et al., 2017) by a researcher blind to treatment and genotype. T_Skin_ was measured by averaging tail temperature in a 1 cm line beginning 2 cm from the tail base. Baseline T_Skin_ measurements were taken immediately following the removal of the cage top and then 7.5, 5, and 2.5 min prior to injection. Following senktide injection, T_Skin_ was measured immediately after injection and every minute for 20 min. Change in T_Skin_ (ΔT_Skin_) due to injection was analyzed, which was calculated as T_Skin_ Raw – T_Skin_ Baseline, where T_Skin_ Raw was the T_Skin_ quantification at a given time point, and T_Skin_ Baseline was the T_Skin_ at 2.5 min prior to injection, which allowed changes in temperature to be normalized to each mouse’s own pre-injection temperature (Krull et al., 2017; Krajewski-Hall et al., 2017).

### 2.8 Uterine weights

Approximately 3 weeks after vasomotor testing, mice were weighed for one final time and euthanized via cervical dislocation. The uterus was immediately dissected and weighed (g).

### 2.9 Data analysis

All statistical analyses were conducted using GraphPad Prism 10 software (La Jolla, CA) as per our previously published methods (Fleischer et al., 2021; Taxier et al., 2022a). To assess within-group learning for OR and OP, one-sample *t*-tests were used to determine whether the time spent with each object during testing significantly differed from chance (15 s). Between-group differences in memory, all EPM measures, and uterine weights were assessed using two-way ANOVAs with treatment and genotype as between-subject variables. T_Skin_ and body weight measures were analyzed using three-way ANOVAs with the same two between-subject variables and time as the within-subject variable. Significant main effects were followed by Tukey’s *post hoc* tests for OR, OP, open field, and EPM data. For all measures except those dependent on time, outliers were defined as values more than two standard deviations from the mean were excluded from analysis. Statistical significance was set at *p* ≤ 0.05 for all statistical tests, and trends were considered as 0.05 ≤ *p* ≤ 0.10.

During OR training, only 3 mice failed to reach the 30 s exploration criterion (one E3FAD treated with vehicle, one E3FAD treated with EGX358, and one E3/4FAD mouse treated with vehicle) and only two mice did so during OP training (the same two E3FAD mice as in OR). When retrained, each mouse reached the 30 s exploration criterion so was advanced to testing and was included in all OR and OP analyses.

## 3 Results

### 3.1 EGX358 improved object recognition memory in E3FAD and E3/4FAD mice

#### 3.1.1 Object recognition

**Figure 2:**
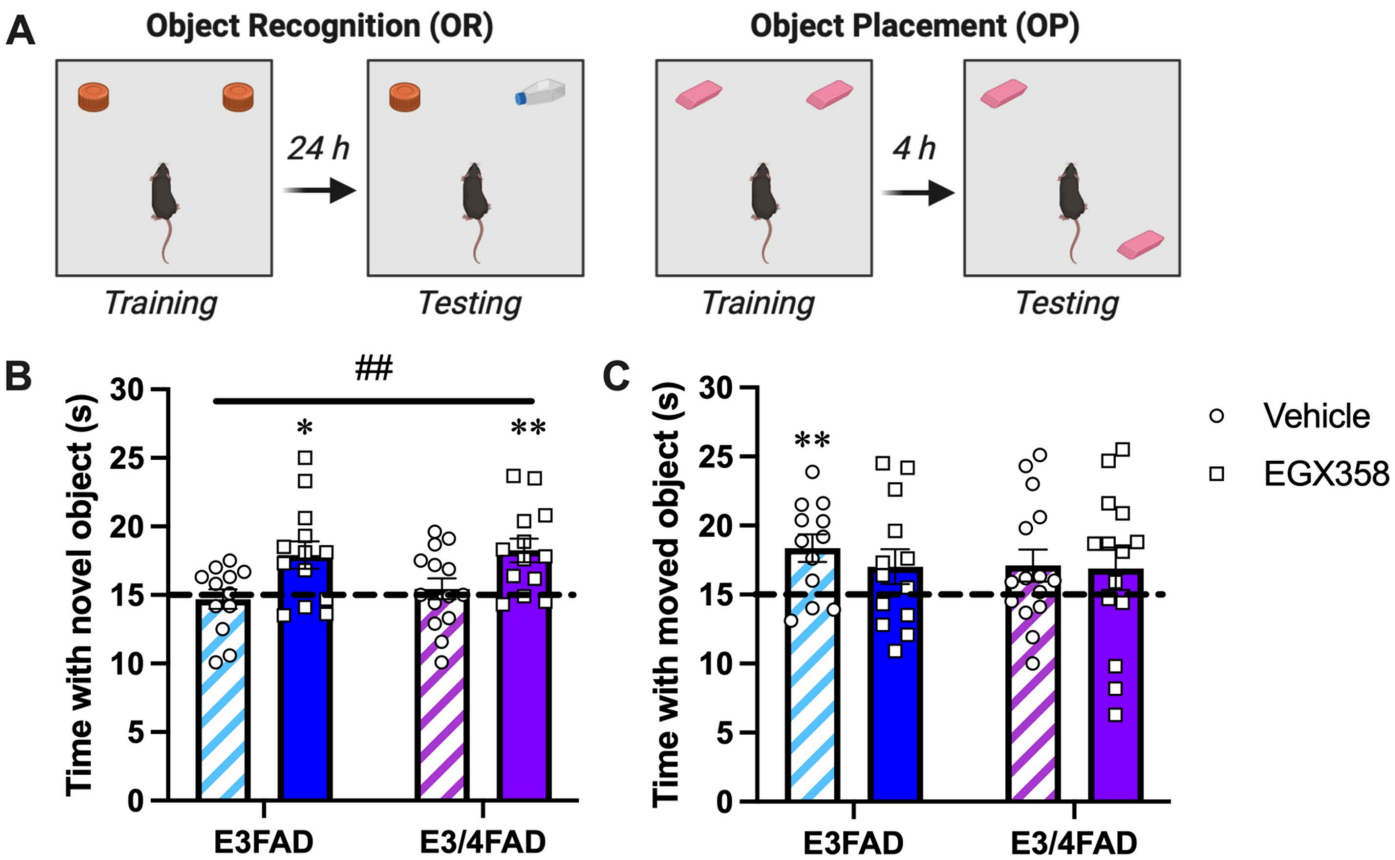
EGX358 improved object recognition memory in E3FAD and E3/4FAD mice but had no effect on object placement. **(A)** Schematic illustration of the object recognition (OR) and object placement (OP) tasks. Figure created in BioRender.com. **(B)** E3FAD and E3/4FAD mice treated with EGX358 spent significantly more time than chance (dashed line at 15 s; *p < 0.05, **p < 0.01 relative to chance) with the novel object during OR testing, whereas vehicle-treated E3FAD and E3/4FAD mice did not, suggesting that EGX358 enhanced OR memory in both genotypes. This conclusion was supported by a significant main effect of Treatment for OR (##*p* < 0.001), which indicates that EGX358 enhanced memory for object identity in both genotypes relative to controls. **(C)** In contrast to OR, vehicle-treated E3FAD females were the only group to spend significantly more time than chance (**p < 0.01) with the moved object during OP testing. EGX358 had no beneficial effects on spatial memory in either genotype. In both panels, bars represent the group mean ± standard error of the mean (SEM). *N* = 12–15/group.

Based upon our previous findings that daily oral gavage of the highly-selective ERβ agonist EGX358 enhanced OP and OR memory in OVX wild-type mice (Fleischer et al., 2021), we sought to determine whether EGX358 might also improve OP and OR memory (Figure 2A) in OVX E3FAD and E3/4FAD females. Here, EGX358 (10 mg/kg/day) was delivered via hydrogel to minimize stress of daily handling and gavage. EGX358 enhanced OR memory in both E3FAD and E3/4FAD mice (Figure 2B) relative to vehicle mice of both genotypes, as indicated by a significant main effect of Treatment (*F*_(1,48)_ = 12.73, *p* = 0.0008) in the absence of a significant main effect of Genotype (*F*_(1,48)_ = 0.4149, *p* = 0.5226) or Treatment x Genotype interaction (*F*_(1,48)_ = 0.0595, *p* = 0.8083). Posthoc Tukey’s multiple comparisons indicated a difference between vehicle- and EGX358-treated E3FAD females (*p* = 0.05) and a trend for a difference between vehicle- and EGX358-treated E3/4FAD females (*p* = 0.0914). The beneficial effects of EGX358 on OR memory in mice of both genotypes were also confirmed by within-group one-sample *t*-tests indicating that only E3FAD (*t*_(12)_ = 2.94, *p* = 0.0124) and E3/4FAD (*t*_(12)_ = 3.742, *p* = 0.0028) females treated with EGX358 spent significantly more time with the novel object than chance (15 s). Vehicle-treated E3FAD (*t*_(11)_ = 0.433, *p* = 0.6734) and E3/4FAD (*t*_(13)_ = 0.578, *p* = 0.5732) females spent chance amounts of time with the two objects, indicating poor memory for the training objects.

Neither treatment nor genotype affected the time to accumulate 30 s of exploration, distance traveled, or velocity in OR (see Table 1 for group means).

**Table 1:** Group means for non-mnemonic measures recorded during object recognition and object placement testing.

Task	Measure	E3FAD		E3/4FAD	
		Vehicle	EGX358	Vehicle	EGX358
Object recognition	Time to accumulate 30s of exploration (s)	605.98 ± 48.29	619.59 ± 50.78	605.63 ± 41.07	613.00 ± 45.37
	Distance traveled (m)	35.30 ± 5.21	35.65 ± 4.99	29.51 ± 2.92	37.85 ± 4.47
	Average velocity (m/s)	0.06 ± 0.01	0.06 ± 0.01	0.05 ± 0.003	0.07 ± 0.07
Object placement	Time to accumulate 30s of exploration (s)^a^	**542.45 ± 42.74**	**625.96 ± 57.08**	**562.54 ± 38.38**	**724.69 ± 49.93**
	Distance traveled (m)	36.95 ± 5.97	34.93 ± 57.08	29.31 ± 3.04	38.56 ± 3.73
	Average velocity (m/s)	0.07 ± 0.01	0.06 ± 0.01	0.05 ± 0.003	0.05 ± 0.003

a Main effect of treatment, *p* < 0.05. Bold values highlight significant treatment effect.

#### 3.1.2 Object placement

In contrast to OR, EGX358 did not facilitate spatial memory in the OP task among EFAD mice of either genotype (Figure 2C). Oddly, one-sample *t*-tests indicated that vehicle-treated E3FAD mice spent significantly more time than chance with the moved object (*t*_(11)_ = 3.358, *p* = 0.0064), which is contrary to our previous work in which OVX or gonadally-intact E3FAD females showed no preference for the moved object 24 h after training (Taxier et al., 2022a,b). More importantly, however, no other group, including both EGX358-treated groups, showed significant preferences for the moved object. Supporting the overall lack of Treatment or Genotype effects on OP, two-way ANOVA indicated no significant effects of Treatment (*F*_(1,50)_ = 0.377, *p* = 0.542), Genotype (*F*_(1,50)_ = 0.3011, *p* = 0.5856), or Treatment x Genotype interaction (*F*_(1,50)_ = 0.19, *p* = 0.664).

Mice treated with EGX358 took more time to accumulate 30 s of exploration during OP testing than those treated with vehicle (*F*_(1,51)_ = 6.607, *p* = 0.0131). Genotype did not affect time to accumulate 30 s (Table 1). Neither distance traveled nor velocity were affected by treatment or genotype in OP (see Table 1 for group means).

### 3.2 *APOE4* genotype, but not EGX358 treatment, reduced anxiety-like behavior in the open field test

We previously showed that OVX E3FAD and E3/4FAD females did not differ in open field measurements of anxiety-like behavior or locomotor activity (Taxier et al., 2022c) and that EGX358 treatment did not affect anxiety-like behaviors in the open field or EPM in OVX wild-type mice (Fleischer et al., 2021). Nevertheless, to measure possible effects of EGX358 in E3FAD and E3/4FAD on anxiety or activity, we used the open field and EPM to measure anxiety-like and exploratory behaviors.

#### 3.2.1 Open field

Data from the first day of habituation to the object box were analyzed as an open field test to provide information about locomotor activity (distance traveled, speed) and anxiety-like behavior (time spent in the center and outer zones). Less time spent in the center zone and more time spent in the outer zone indicated higher anxiety-like behavior.

**Figure 3:**
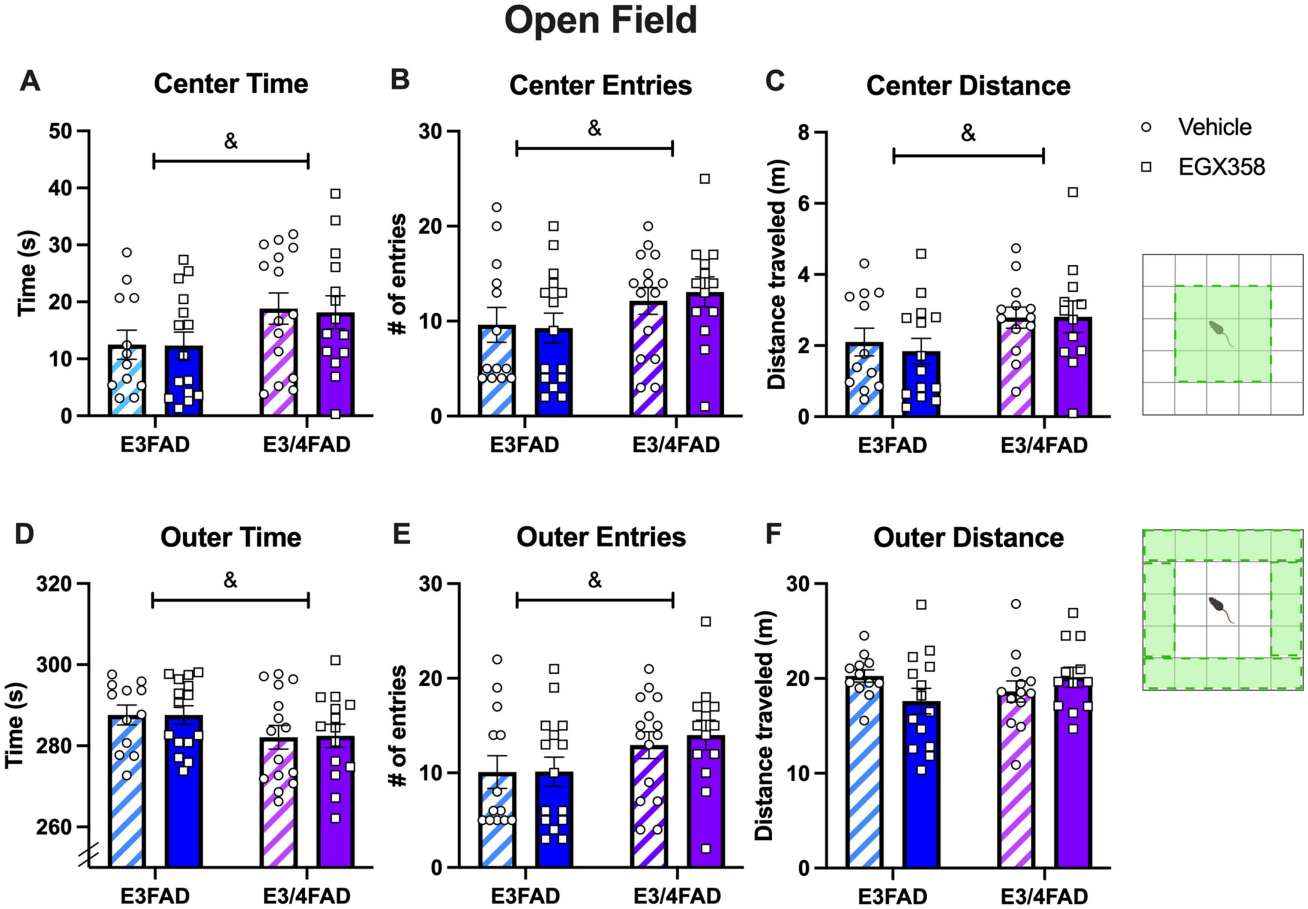
E3/4FAD mice exhibited fewer anxiety-like behaviors than E3FAD mice, regardless of treatment. **(A–C)** E3/4FAD females spent more time in the center zone of the open field, entered the center zone more, and traveled more distance in the center than E3FAD females (& = main effect of Genotype, *p* < 0.05). **(D–F)** E3/4FAD mice spent less time in the outer zone, yet made more entries into this zone, although the distance traveled in the outer zone did not differ by genotype (& = main effect of Genotype, *p* < 0.05). Treatment had no effect on any open field measure. In both panels, bars represent the group mean ± SEM, *N* = 12–15/group in all panels. Inset diagrams to the right illustrate the center and outer zones. Open field schematics to the right of the figure were created in BioRender.com.

E3/4FAD females spent significantly more time in the center zone (*F*_(1,52)_ = 5.183, *p* = 0.027; Figure 3A) and made more entries into the center zone (*F*_(1,52)_ = 3.914, *p* = 0.05; Figure 3B) than E3FAD females while spending significantly less time in the outer zone (*F*_(1,52)_ = 3.961, *p* = 0.05; Figure 3D) and making fewer entries into the outer zone (*F*_(1,52)_ = 4.609, *p* = 0.0365; Figure 3E) than E3FAD females, suggesting less anxiety-like behavior in the open field in E3/4FAD mice relative to their E3FAD counterparts. However, treatment had no impact on the time (*Fs*_(1,52)_ = 0.0223 and 0.0057, *ps* > 0.05) or entry (*Fs*_(1,52)_ = 0.0346 and 0.1286, *ps* > 0.05) measures, nor were the interactions significant (time: *Fs*_(1,52)_ = 0.0077 and 0.0046, *ps* > 0.05; entries: *Fs*_(1,52)_ = 0.1632 and 0.1041, *ps* > 0.05).

Although E3/4FAD mice also traveled further distance in the center zone than E3FAD mice (*F*_(1,47)_ = 4.895, *p* = 0.0318; Figure 3C), the genotypes traveled similar distances in the outer zone (*F*_(1,47)_ = 0.1637, *p* = 0.6876; Figure 3F) and similar total distance (*F*_(1,47)_ = 1.013, *p* = 0.3194), indicating no genotype effect on overall activity in the arena. Treatment had no impact on any distance measure, as either a main effect or interaction. Moreover, neither treatment nor genotype affected locomotor speed. Thus, locomotor abilities were not affected by *APOE* genotype or treatment, indicating that these results were likely not due to systemic physiological effects of *APOE* status or treatment with EGX358.

#### 3.2.2 Elevated plus maze

The EPM was used as a second measure of anxiety-like behavior. Number of entries into and time spent in the open arms, closed arms, and center zone were quantified. Neither treatment nor genotype significantly affected any of these measures (see Table 2 for group means).

**Table 2:** Group means for elevated plus maze measures.

Measure	E3FAD		E3/4FAD	
	Vehicle	EGX358	Vehicle	EGX358
# entries into open arms	1.92 ± 0.34	3.00 ± 0.75	2.36 ± 0.45	2.31 ± 0.51
Time in open arms (s)	12.81 ± 3.18	22.45 ± 6.65	16.60 ± 3.38	16.99 ± 4.04
# entries into center	32.92 ± 2.78	37.36 ± 2.23	29.86 ± 1.97	32.77 ± 1.76
Time in center (s)	131.09 ± 6.93	141.77 ± 4.45	118.62 ± 9.02	130.92 ± 6.57
# entries into closed arms	31.33 ± 2.69	34.64 ± 2.51	27.71 ± 1.68	30.92 ± 1.62
Time in closed arms (s)	454.99 ± 9.13	435.01 ± 6.61	463.32 ± 11.30	453.82 ± 7.65

### 3.3 *APOE4* heightened the vasomotor response to a NK3R agonist, but this effect was not mitigated by EGX358

**Figure 4:**
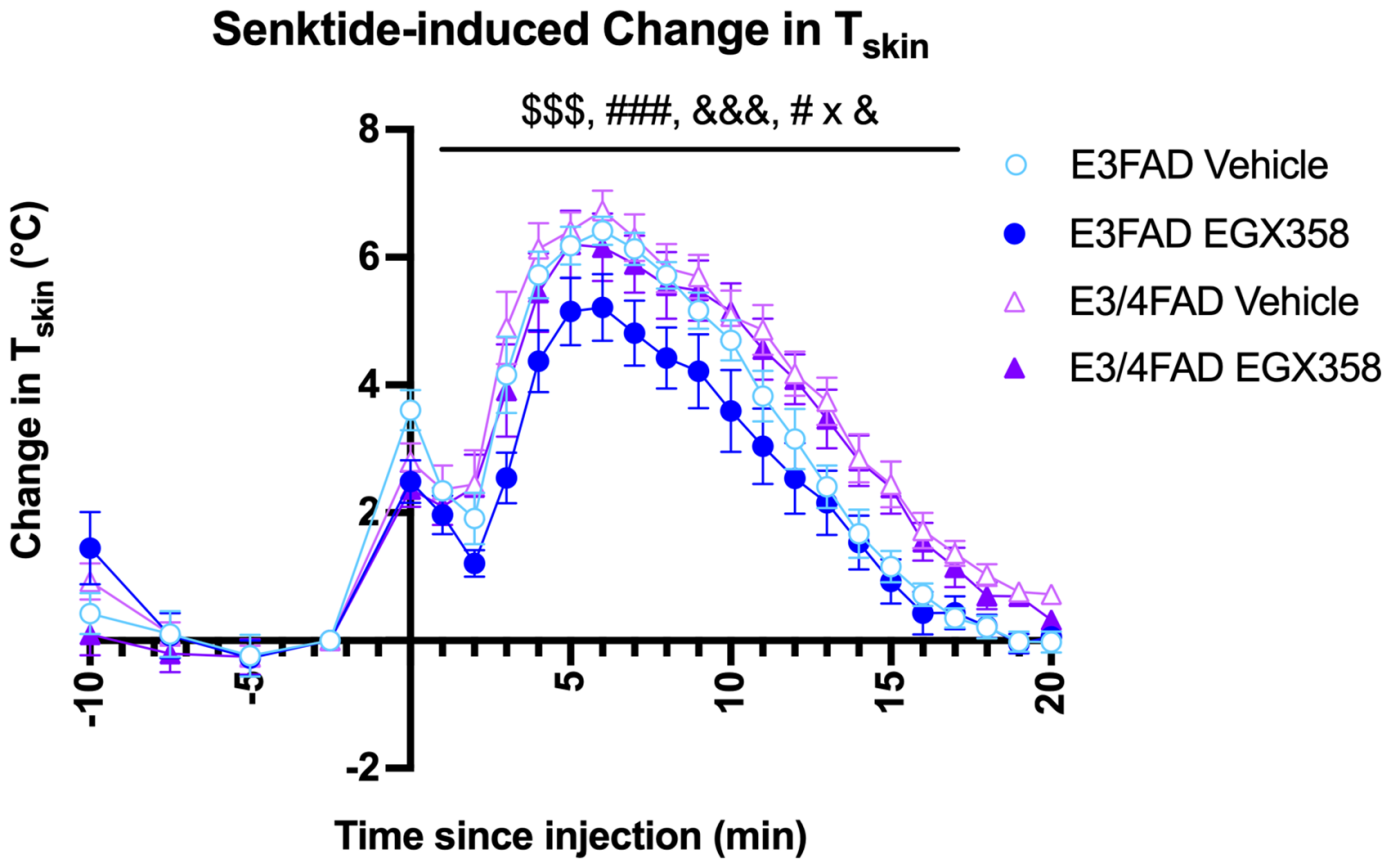
EGX358 reduced the magnitude of a drug-induced hot flash in E3FAD females only. Senktide injection (time 0, x-axis) significantly increased T_Skin_ by about 4^o^ in all groups within 5 min, followed by a gradual return to baseline by about 20 min ($$$ = main effect of Time, *p* < 0.0001). EGX358 treatment significantly reduced the magnitude of the increase in T_Skin_ among E3FAD females only (### = main effect of Treatment, *p* < 0.0001; &&& = main effect of Genotype, *p* < 0.0001; # x & = Treatment x Genotype interaction, *p* < 0.05). Numbers to the left of the y-axis represent baseline measurements in the 10 min before senktide injection and are therefore not included in the analysis of senktide response. Symbols represent the group mean ± SEM, *N* = 13–15/group.

Because we previously found that long-term oral EGX358 administration reduced the magnitude of change in T_Skin_ in response to the NK3R agonist senktide in OVX wild-type C57BL/6 mice (Fleischer et al., 2021), we next assessed the extent to which *APOE* genotype might affect the ability of EGX358 to alleviate this drug-induced hot flash. Senktide caused a rapid and transient increase in T_Skin_, such that T_Skin_ in all groups was significantly increased within 5 min and then returned to baseline within 15 min (*F*_(20, 1,071)_ = 106.4, *p* < 0.0001; Figure 4). This increase was especially pronounced in E3/4FAD females compared to E3FAD females (*F*_(1, 1,071)_ = 92.11, *p* < 0.0001) and in vehicle-treated mice relative to those treated with EGX358 (*F*_(1, 1,071)_ = 32.47, *p* < 0.0001). As illustrated in Figure 4, EGX358 significantly reduced the magnitude of the increase in T_Skin_ in E3FAD females but not in E3/4FAD females (Genotype x Treatment; *F*_(1, 1,071)_ = 5.466, *p* = 0.0196).

### 3.4 E3/4FAD mice gained more weight following ovariectomy, whereas EGX358 slightly reduced uterine atrophy

#### 3.4.1 Body weights

**Figure 5:**
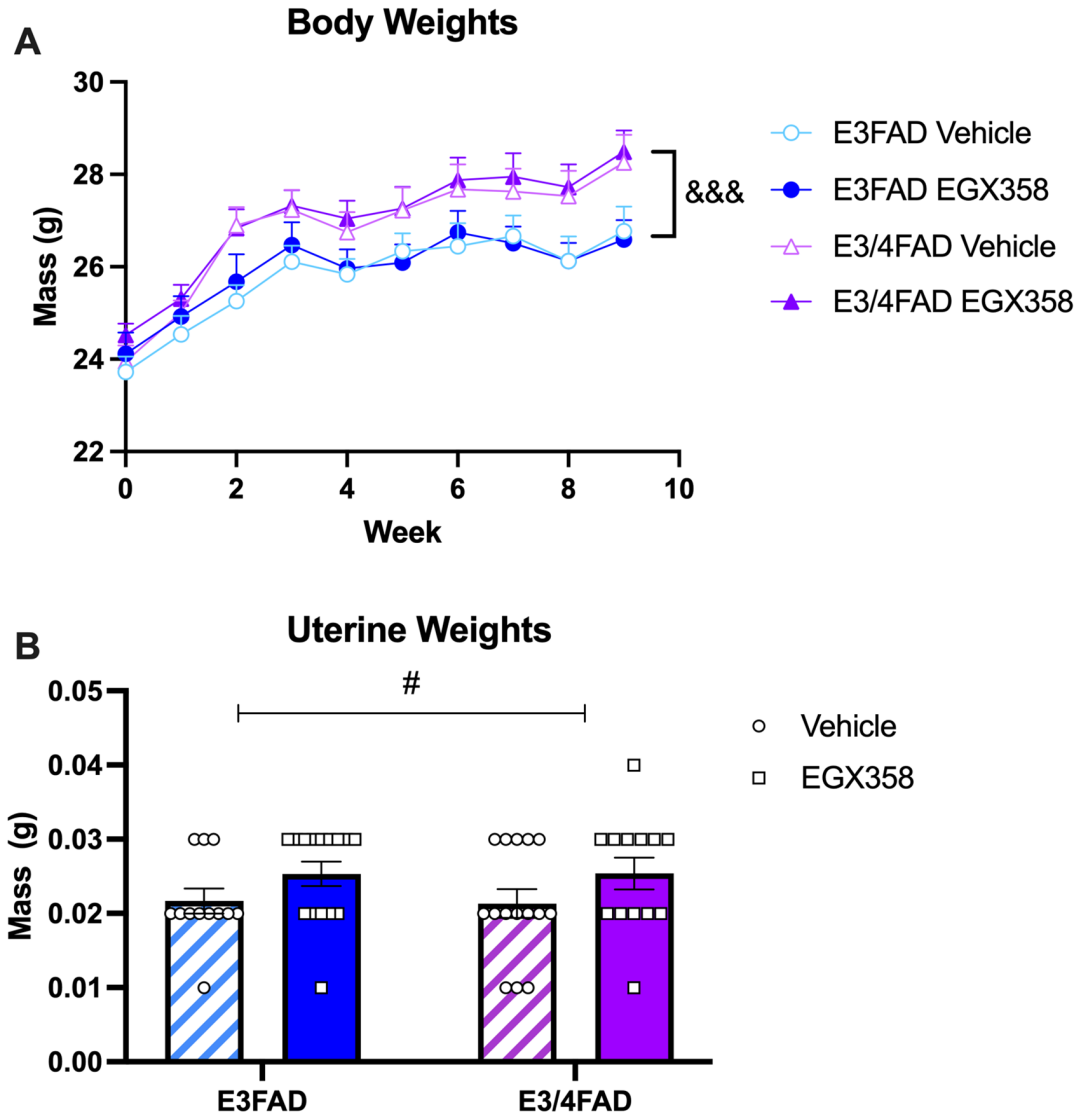
E3/4FAD mice gained more weight regardless of treatment; EGX358 increased uterine weights in both genotypes. **(A)** Although all groups gained weight after ovariectomy, E3/4FAD females gained more weight throughout the study than E3FAD females, regardless of treatment (&&& = main effect of Genotype, *p* < 0.0001). **(B)** By the end of the study, uterine weights were significantly higher in EGX358-treated mice of both genotypes (# = main effect of Treatment, *p* < 0.05), suggesting that EGX358 mitigated some of the uterine atrophy induced by ovariectomy. Symbols and bars represent the group mean ± SEM, *N* = 13–15 in both panels.

Mice were weighed weekly throughout the experiment to analyze potential effects of genotype and treatment on body weight over time (Figure 5A). All groups gained weight throughout the course of the study (*F*_(1.47, 78)_ = 96.96, *p* < 0.0001). Interestingly, E3/4FADs gained significantly more weight over the course of the study than E3FADs (Genotype: *F*_(1, 53)_ = 7.565, *p* = 0.0081; Genotype x Time: *F*_(9, 477)_ = 3.918, *p* < 0.0001). However, EGX358 did not influence body weight, as there was no significant main effect of treatment or treatment-related interactions on body weight.

#### 3.4.2 Uterine weights

At the time of tissue collection, uteri were removed and weighed to the nearest 0.01 g (Figure 5B). There was a small, but statistically significant increase in uterine weights in EGX358-treated mice compared to vehicle (*F*_(1, 51)_ = 4.247, *p* = 0.0444). Given the low magnitude of this increase (~0.005 g), and low average weight compared to previously published data from gonadally intact mice (~ 0.125 g) (Modder et al., 2004), this EGX358-induced increase cannot be considered hypertrophy. Genotype did not affect uterine weights, either as a main effect (*F*_(1, 51)_ = 0.0056, *p* = 0.9403) or interaction (*F*_(1, 51)_ = 0.0105, *p* = 0.9403).

## 4 Discussion

### 4.1 EGX358 improved object recognition, but not object placement, in both E3FAD and E3/4FAD females

The primary goal of this study was to determine the extent to which the novel highly selective ERβ agonist EGX358 could enhance object recognition and spatial memory in EFAD mice homozygous for human *APOE3* or heterozygous for human *APOE3* and *APOE4*. We focused on E3FAD and E3/4FAD mice due to previous work from the Frick lab showing that an acute dorsal hippocampal infusion of E_2_ increases CA1 dendritic spine density and enhances memory consolidation in the object recognition and object placement tasks among OVX E3FAD and E3/4FAD, but not E4FAD, mice (Taxier et al., 2022a). Accordingly, subsequent work from the LaDu lab showed that spatial memory in the Morris water maze was also improved by long-term oral administration of E_2_ in OVX E3FAD and E3/4FAD, but not E4FAD, mice (Balu et al., 2024). Thus, we hypothesized that only E3FAD and E3/4FAD females would be responsive to an ERβ agonist like EGX358 in measures of spatial and object recognition memories.

In OVX female wild-type mice and rats, acute systemic injection or dorsal hippocampal infusion of commercial ERβ agonists like diarylpropionitrile (DPN) consistently enhance memory in tasks like object recognition and object placement (Boulware et al., 2013; Tuscher et al., 2015). Moreover, dorsal hippocampal infusion of an ERβ antagonist blocks memory consolidation in both tasks among OVX wild-type mice (Kim and Frick, 2017), suggesting that activation of ERβ in the dorsal hippocampus is both sufficient and necessary for object recognition and spatial memory formation. Consistent with these effects, we previously showed that long-term daily oral administration of EGX358 to OVX wild-type mice enhanced memory in both object tasks and reduced the magnitude of a senktide-induced hot flash to a similar extent as E_2_ and DPN, without affecting anxiety- or depression-like behaviors or body weight (Fleischer et al., 2021). Here, we found that long-term daily oral treatment with EGX358 improved object recognition memory in OVX E3FAD and E3/4FAD mice relative to vehicle controls, which is consistent with our data from wild-type mice. In light of the amyloid burden and synaptic degeneration previously observed in 6 month-old female E3FAD and E3/4FAD mice (Youmans et al., 2012; Tai et al., 2014; Liu et al., 2015; Cacciottolo et al., 2016; Balu et al., 2023), these findings suggest the exciting possibility that ERβ agonist treatment may not only be beneficial for recognition memory in menopausal women without dementia, but also in *APOE3/3+* and *APOE3/4+* women with symptoms of cognitive decline.

In contrast to our previous wild-type findings, EGX358 did not enhance memory in the object placement test relative to chance or to vehicle controls. This null effect could be explained, in part, due to the unexpectedly strong memory shown by the vehicle-treated E3FAD group; in our prior studies, vehicle-treated OVX and gonadally-intact female E3FAD mice did not show evidence of learning in either object task (Taxier et al., 2022a,b). However, comparisons with chance indicate that EGX358 did not significantly increase the time spent with the moved object in either E3FAD or E3/4FAD females, suggesting no benefit to learning within each genotype. This finding contrasts with previously beneficial effects on memory of acute or chronic E_2_ treatments in these genotypes (Taxier et al., 2022a; Balu et al., 2024). The most obvious explanation is that spatial memory as assessed by object placement is not sensitive to ERβ agonism in mice with established AD-like pathologies including high levels of Aβ42, oligomeric Aβ, amyloid deposition, phospho-tau, gliosis, and synaptic degeneration. However, that conclusion is premature, as the effects of treatment could not truly be assessed here due to the performance of vehicle-treated E3FAD mice. In addition, methodological factors like drug dose and method of delivery (hydrogel here vs. gavage in our previous work) may also play a role. Future studies should re-examine effects of EGX358 on OP memory in EFAD mice and address whether spatial memory in OVX E3FAD and E3/4FAD mice might be enhanced by higher doses of EGX358 or a more controlled method of administration (e.g., gavage).

### 4.2 *APOE4* blunted the beneficial effects of EGX358 on vasomotor response

As in our previous work (Fleischer et al., 2021), we modeled a hot flash in this study using the selective neurokinin-3 tachykinin receptor agonist senktide. Senktide mimics the hormone neurokinin B, which is released by kisspeptin, neurokinin B, and dynorphin (KNDy) neurons in the hypothalamus. It causes vasodilation by binding neurokinin-3 tachykinin receptors and activating warm-sensitive neurons in the ventromedial preoptic area, resulting in rapid and transient increases in tail skin temperature in OVX mice and gonadally-intact mice of both sexes (Krull et al., 2017; Krajewski-Hall et al., 2017). In our hands, subcutaneous injection of 0.5 mg/kg senktide in OVX wild-type mice produces a T_Skin_ increase of >3^o^ within 5 min that returns to baseline within about 15 min, and the magnitude of this increase is reduced similarly by E_2_, DPN, and EGX358 (Fleischer et al., 2021). In this study, senktide injection caused a rapid and transient increase in T_Skin_ among mice of both genotypes, although the increase was generally of a somewhat higher magnitude (>4^o^) and lasted longer compared to wild-type mice. Importantly, however, EGX358 treatment reduced the magnitude of the change in T_Skin_ among E3FAD, but not E3/4FAD, mice. These findings suggest that presence of even a single *APOE4* allele is sufficient to block the beneficial effects of long-term ER*β* agonism in a preclinical model of menopause-related hot flashes. This result is consistent with clinical data showing that phytoSERM compounds – plant-derived selective estrogen receptor modulators – that preferentially bind to ERβ, including genistein, daidzein, and S-equol, reduced the frequency of hot flashes in *APOE3+*, but not *APOE3/4+*, women (Wang et al., 2020).

Our results also align with a previous study of metabolic profiles in male mice expressing human *APOE3* or *APOE4*. *APOE4* mice had increased lipid metabolism, increased body temperature, and more metabolically active brown adipose tissue (Arbones-Mainar et al., 2016). A handful of studies have examined neurokinin signaling in rodent models of dementia and found that senktide reversed spatial memory deficits in male Wistar rats infused with Aβ1-42 (Koca et al., 2023), and reduced dentate gyrus circuit disorders in socially-isolated male 3x Tg-AD mice (Huang et al., 2023), possibly due to the promotion of forebrain cholinergic activity by neurokinin 3 signaling (de Souza Silva et al., 2013). However, to the best of our knowledge, this is the first study to examine whether *APOE* genotype affects the vasomotor response to senktide or any NK3 receptor agonist in male or female rodent models of AD. In a small observational study in middle-aged women, hot flashes were linked to worse verbal memory, and the degree of impairment may have been affected by secondary symptoms, such as worsening sleep quality (Maki et al., 2008). More recently, higher levels of objectively assessed vasomotor symptoms, particularly at night, were associated with higher plasma biomarkers of amyloid plaques (Thurston et al., 2024). Thus, the *APOE* genotype differences observed here in response to senktide injection suggest a critical need to more fully examine how symptoms associated with the menopausal transition, such as hot flashes, may contribute to cognitive decline and cognitive response to estrogen therapy.

### 4.3 *APOE4* had modest anxiolytic effects irrespective of EGX358 treatment

We also examined anxiety-like behaviors in the open field and elevated plus maze, given a previous report from the Frick lab that EFAD females with two copies of *APOE4* (E4FAD) exhibited greater levels of anxiety-like behavior in the open field (less time in the center) relative to E3FAD females (Taxier et al., 2022c). Thus, we thought it possible that E3/4FAD females might be more anxious than E3FAD females. Although ER*β* agonism has been reported to lower anxiety-related phenotypes in mice and rats (Rocha et al., 2005; Walf and Frye, 2005; Walf and Frye, 2007), other studies report no effects of ERβ agonism on anxiety- and depression-like behaviors in female mice (Eid et al., 2020). Likewise, long-term oral gavage of EGX358 did not alter exploratory behaviors in the open field or EPM tests among wild-type mice (Fleischer et al., 2021), so we did not expect treatment effects in either genotype. Consistent with this hypothesis, EGX358 did not affect any measure of open field or elevated plus maze behavior. However, we surprisingly found that E3/4FAD mice spent *more* time in the center of the open field than their E3FAD counterparts, irrespective of treatment, suggesting less anxiety-like behavior in mice with one copy of *APOE4*. This result is somewhat perplexing given that two copies of *APOE4* previously reduced center time in the open field (Taxier et al., 2022c). Interestingly, neither treatment nor genotype affected activity in the elevated plus maze, another measure of anxiety-like behavior, so the extent to which *APOE* genotype more generally modulates anxiety-like and exploratory behaviors is unclear.

It should be noted that we took great care in our treatment and handling protocols to minimize chronic stress in our mice, which perhaps partially explains the modest anxiety-related results. In human studies, *APOE4* carriers are more likely to have anxiety than non-carriers (Holmes et al., 2016; Xu et al., 2023), so our results are unexpected in this light. Regarding EGX358, the lack of effects in the open field and plus maze could indicate that ERβ is not involved in regulating anxiety-like behaviors among individuals with established brain amyloid, phospho-tau burden, and AD-related neurodegeneration, and may not be an effective therapy for mood-related symptoms in AD patients. However, considerably more work will be needed to fully understand the clinical applicability of these compounds for anxiety- and depression-related behaviors in AD patients. Critically, EGX358 did not produce any adverse effects on anxiety-like behaviors in either of our tests, suggesting that it could be used as a therapeutic for memory and hot flashes without negatively impacting anxiety.

### 4.4 *APOE4* increased body weights irrespective of EGX358 treatment

Estrogens such as E_2_ regulate energy balance and homeostasis, and their loss at menopause and after OVX in rodents is associated with increased fat mass and body weight (Mauvais-Jarvis et al., 2013; Palacios et al., 2024; Roesch, 2006). Thus, we assessed the effects of EGX358 treatment on body weights throughout the experiment. As in our previous work with long-term oral EGX358 treatment in wild-type female mice (Fleischer et al., 2021), EGX358 in this study did not influence body weights throughout the 10 weeks of treatment. This null effect is consistent with studies showing that neither long-term ERβ agonist treatment nor knockout of ERβ prevent OVX-induced increases in body weights in rodents (Roesch, 2006; Opas et al., 2009; Van Pelt et al., 2015). Indeed, studies of knockout mice and ER*α* agonists indicate that the effects of estrogens on body weight are largely driven by ERα (Roesch, 2006; Van Pelt et al., 2015; Wegorzewska et al., 2008), so the inability of EGX358 to counter the lipogenic effects of OVX are not surprising. Nevertheless, the present data are an important confirmation of our earlier work that long-term oral EGX358 treatment has no adverse effects on body weight, which suggests that EGX358 treatment in humans would not likely cause undue weight gain or loss.

In contrast to treatment, *APOE* genotype significantly affected body weights. Although weekly body weights increased throughout the study in all groups, as would be expected after OVX, E3/4FAD mice in both treatment groups gained significantly more weight during the course of the experiment than both E3FAD groups. All groups started at approximately the same mean body weight, but weights increased nearly two grams in both E3/4FAD groups during week 2 and this genotype difference continued for the remainder of the study. A recent study in young adult female E3FAD and E4FAD mice found that E4FADs actually gained slightly less weight over the course of the 12 week study, but had a higher percentage of visceral body fat (Christensen and Pike, 2023). Additionally, E4FADs had higher blood levels of insulin, leptin, and total cholesterol, which are associated with metabolic impairments (Christensen and Pike, 2023). Another study in *APOE* knock-in mice found no effect of genotype in females on weight gain over the 12 week study, both in high-fat and low-fat diets (Jones et al., 2019). Because all mice used in our study were ovariectomized, *APOE4* may have amplified the effects of ovarian hormone loss on metabolism and adiposity, causing OVX E3/4FADs to gain more weight than E3FADs. In humans, hypothalamic atrophy in AD patients is thought to lead to insulin and leptin resistance, ultimately leading to weight loss as the disease progresses (Ishii and Iadecola, 2015; Vercruysse et al., 2018). This loss occurs earlier and to a greater extent in AD patients that express at least one copy of *APOE4*, particularly women *APOE4* carriers (Ukraintseva et al., 2024; Ando et al., 2022; Vanhanen et al., 2001). Thus, the weight gain observed in E3/4FAD mice is not consistent with clinical findings. Although OVX is an imperfect model of menopause, the abrupt cessation of circulating ovarian hormones following surgery has repeatedly been shown to mimic the menopause-associated gain in weight, and estrogen treatments consistently ameliorate this weight gain both in women and OVX female rodents (Witte et al., 2010; Mamounis et al., 2014). However, widespread brain alterations in AD must impact the interplay between metabolic function and gonadal hormones in ways that differ from aging or menopause alone, so future studies will be needed to better understand the disparate effects of OVX on body weight in E3/4FAD mice compared to menopause in *APOE4*+ women with AD.

### 4.5 EGX358 somewhat mitigated ovariectomy-induced uterine weight atrophy

Finally, we measured effects of EGX358 treatment on uterine weights given the well-known uterotrophic effects of estrogen treatments (Modder et al., 2004; Zhu and Pollard, 2007). Although a significant main effect of treatment was observed, such that EGX358 treatment was associated with increased uterine weights, all weights remained considerably less than those of gonadally-intact C57BL/6 mice (typically ~150 mg). In one study in which uterine weights of sham-operated C57BL/6 females averaged 150 mg, OVX reduced uterine weights to a mean of 50 mg and 60 days of E_2_ treatment dose-dependently increased weights to between 150–200 mg (Modder et al., 2004). As such, the EGX358-induced increase here, although statistically significant, is not reflective of uterine hypertrophy. Indeed, this increase somewhat mitigates the atrophic state of the uterus after ovary removal, which may be beneficial for uterine health.

## 5 Conclusion

In conclusion, the results of the present study indicate the potential for selective ER*β* agonism to improve recognition memory and alleviate hot flashes in females with established AD-like brain pathology without adverse effects on anxiety-like behaviors, body weight, and uterine weight. Beneficial effects were particularly evident for E3FAD mice, with improvements observed for object recognition memory and mitigation of a drug-induced hot flash. *APOE3* is the most common *APOE* allele and although *APOE4* is more associated with AD, a substantial number of AD patients are *APOE4*- (Heffernan et al., 2016; Farrer et al., 1997). As such, the fact that EGX358 could alleviate memory and vasomotor deficits in E3FAD mice suggests potentially promising efficacy for EGX358 in *APOE4*- women with AD. However, *APOE4* status is an important mitigating factor that must be taken into account when considering treatment options, as this allele blocked the effects of EGX358 treatment on vasomotor symptoms and influenced body weight and some anxiety-like behaviors. Nevertheless, EGX358 did improve object recognition memory in E3/4FAD mice, so could potentially alleviate memory loss in some *APOE*3/4 individuals. Future work should also determine potential efficacy in *APOE4/4* individuals, as targeted agonism of ERβ could provide greater benefits than the broader ER activation produced by traditional hormone therapies. For now, however, the present study suggests that highly selective ERβ agonists such as EGX358 may be a promising therapeutic option for individuals with AD that warrants further preclinical testing.

## Data Availability

The raw data supporting the conclusions of this article will be made available by the authors, without undue reservation.

## In memoriam

We dedicate this article to our colleague Dr. Mary Jo LaDu, who passed away much too soon in March 2023. She was dedicated to understanding how *APOE*, sex, and age interact to increase women’s risk of AD and embraced estrogens as potential preventative and restorative treatments. The EFAD model is her gift and legacy to the AD field.
